# Older patients with COVID‐19 and neuropsychiatric conditions: A study of risk factors for mortality

**DOI:** 10.1002/brb3.2787

**Published:** 2022-11-10

**Authors:** Vi‐Huong Nguyen‐Michel, Marion Houot, Cécile Delorme, Aude Sangaré, Ana Gales, Valerio Frazzini, Aurélie Hanin, Djamal Aissani, Thanh Trân, Bruno Oquendo, Flora Ketz, Carmelo Lafuente‐Lafuente, Christel Oasi, Kiyoka Kinugawa, Gaëlle Ouvrard, Renata Ursu, Bertrand Degos, Benjamin Rohaut, Sophie Demeret, Virginie Lambrecq, Vincent Navarro, Emmanuel Fournier, Jean‐Christophe Corvol, Alaina Borden

**Affiliations:** ^1^ Sorbonne Université, AP‐HP, Pitié‐Salpêtrière Charles‐Foix Hospital Group, Clinical Neurophysiology Department, EEG‐Epilepsy Unit Functional Exploration Unit for the Older Patients Paris France; ^2^ Sorbonne Université, AP‐HP Pitié‐Salpêtrière Hospital, Epileptology Unit Paris France; ^3^ Sorbonne Université Paris Brain Institute – ICM, INSERM, CNRS Paris France; ^4^ Sorbonne Université, AP‐HP, Pitié‐Salpêtrière Hospital Institut de la Mémoire et de la maladie d'Alzheimer Paris France; ^5^ Sorbonne Université, AP‐HP, Pitié‐Salpêtrière Hospital Center of Excellence of Neurodegenerative Disease (CoEN) Paris France; ^6^ Sorbonne Université, AP‐HP, Pitié‐Salpêtrière Hospital Department of Neurology Paris France; ^7^ Sorbonne Université, AP‐HP, Pitié Salpêtrière Hospital Sleep Disorders Unit Paris France; ^8^ Sorbonne Université, AP‐HP, Pitié‐Salpêtrière Charles‐Foix Hospital Group Department of Radiology Paris France; ^9^ Pierre Bérégovoy Hospital Neurological Unit Nevers France; ^10^ Sorbonne Université, AP‐HP, Charles‐Foix Hospital Geriatric Department Paris France; ^11^ Sorbonne Université, CNRS UMR 8256 Biological Adaption and Aging Paris France; ^12^ Sorbonne Université, AP‐HP, Rothschild Hospital Neurological Rehabilitation Unit Paris France; ^13^ Université de Paris, AP‐HP Nord, Saint‐Louis Hospital Neurological Unit Paris France; ^14^ Sorbonne Université Paris Nord, AP‐HP, Avicenne Hospital Department of Neurology Bobigny France; ^15^ Sorbonne Université, AP‐HP, Pitié‐Salpêtrière Hospital Neurological Intensive Care Unit Paris France

**Keywords:** COVID‐19, mortality, neurology psychiatric, older people

## Abstract

**Background:**

Little is known about risk factors for mortality in older patients with COVID‐19 and neuropsychiatric conditions.

**Methods:**

We conducted a multicentric retrospective observational study at Assistance Publique‐Hôpitaux de Paris. We selected inpatients aged 70 years or older, with COVID‐19 and preexisting neuropsychiatric comorbidities and/or new neuropsychiatric manifestations. We examined demographics, comorbidities, functional status, and presentation including neuropsychiatric symptoms and disorders, as well as paraclinical data. Cox survival analysis was conducted to determine risk factors for mortality at 40 days after the first symptoms of COVID‐19.

**Results:**

Out of 191 patients included (median age 80 [interquartile range 74–87]), 135 (71%) had neuropsychiatric comorbidities including cognitive impairment (39%), cerebrovascular disease (22%), Parkinsonism (6%), and brain tumors (6%). A total of 152 (79%) patients presented new‐onset neuropsychiatric manifestations including sensory symptoms (6%), motor deficit (11%), behavioral (18%) and cognitive (23%) disturbances, gait impairment (11%), and impaired consciousness (18%). The mortality rate at 40 days was 19.4%. A history of brain tumor or Parkinsonism or the occurrence of impaired consciousness were neurological factors associated with a higher risk of mortality. A lower Activities of Daily Living score (hazard ratio [HR] 0.69, 95% confidence interval [CI] 0.58–0.82), a neutrophil‐to‐lymphocyte ratio ≥ 9.9 (HR 5.69, 95% CI 2.69–12.0), and thrombocytopenia (HR 5.70, 95% CI 2.75–11.8) independently increased the risk of mortality (all *p* < .001).

**Conclusion:**

Understanding mortality risk factors in older inpatients with COVID‐19 and neuropsychiatric conditions may be helpful to neurologists and geriatricians who manage these patients in clinical practice.

## Graphical abstract

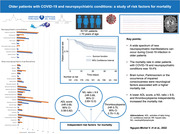

## 1 INTRODUCTION

Advanced age is associated with a high risk of coronavirus disease 2019 (COVID‐19) and mortality (Abbatecola & Antonelli‐Incalzi et al., 2020; Cummings et al., 2020; Richardson et al., 2020). Several studies have focused on older adults with COVID‐19 (Annweiler et al., 2021; Atkins et al., 2020; Covino et al., 2021; Graham et al., 2020; Hägg et al., 2020; Karlsson et al., 2021; Knopp et al., 2020; Li et al., 2020; Miles et al., 2020; Neumann‐Podczaska et al., 2020; Ramos‐Rincon et al., 2021; Steinmeyer et al., 2020; Sun et al., 2020; Vrillon et al., 2020, 2021; L. Wang et al., 2020; Zerah et al., 2021), but few studies have specifically examined risk factors for mortality in a population of older people with Covid‐19 and with neuropsychiatric disorders. Indeed, these studies did not always mention neuropsychiatric comorbidities and provided little insight into new‐onset neuropsychiatric manifestations during the course of COVID‐19. The relationships between mortality in older patients with COVID‐19 and preexisting dementia (Atkins et al., 2020; Covino et al., 2021; De Smet et al., 2020; Genet et al., 2020; Graham et al., 2020; Mendes et al., 2020; Neumann‐Podczaska et al., 2020; Ramos‐Rincon et al., 2021; Steinmeyer et al., 2020; Vrillon et al., 2020; Zerah et al., 2021) or a history of stroke (Atkins et al., 2020; Covino et al., 2021; Genet et al., 2020; Graham et al., 2020; Mendes et al., 2021, 2020; Neumann‐Podczaska et al., 2020; Vrillon et al., 2020; L. Wang et al., 2020; Zerah et al., 2021) have been studied, but little is known about Parkinsonism (Mendes et al., 2020; Vrillon et al., 2021; Zerah et al., 2021), depression (Atkins et al., 2020; Genet et al., 2020; Vrillon et al., 2020), brain tumors, epilepsy, as well as other preexisting neuropsychiatric disorders in older patients. Certain new‐onset neurological symptoms or disorders (and their relationship with mortality for some studies) have been examined in older patients with COVID‐19 including anosmia, ageusia, myalgia (Annweiler et al., 2021; Covino et al., 2021; Mendes et al., 2020; Neumann‐Podczaska et al., 2020; Steinmeyer et al., 2020; Vrillon et al., 2020; L. Wang et al., 2020; Zerah et al., 2021), headache (Covino et al., 2021; Mendes et al., 2020; Steinmeyer et al., 2020; Vrillon et al., 2020, 2021; L. Wang et al., 2020; Zerah et al., 2021), confusion/delirium (Annweiler et al., 2021; Covino et al., 2021; Graham et al., 2020; Karlsson et al., 2021; Knopp et al., 2020; Ramos‐Rincon et al., 2021; Steinmeyer et al., 2020; Vrillon et al., 2020, 2021; Zerah et al., 2021), stroke (Ramos‐Rincon et al., 2021; Vrillon et al., 2021; Zerah et al., 2021), seizures (Mendes et al., 2021; Ramos‐Rincon et al., 2021; Vrillon et al., 2021), and encephalopathy (Martín‐Jiménez et al., 2020), but the spectrum of neurological and psychiatric complications was found to be much larger in adult patients than previously thought (Delorme et al., 2021; Frontera et al., 2021; Mao et al., 2020; Zhao et al., 2020). In older patients, studies examining a large spectrum of neuropsychiatric manifestations during COVID‐19 are lacking. Consequently, little is known about risk factors for mortality in older patients with COVID‐19 and neuropsychiatric conditions.

Here, we examined the risk factors for mortality in hospitalized patients aged 70 years or older, with COVID‐19 and neuropsychiatric comorbidities and or new neuropsychiatric manifestations, taking into account general, geriatric, and paraclinical findings.

## 2 METHODS

### 2.1 Participants

**FIGURE 1:**
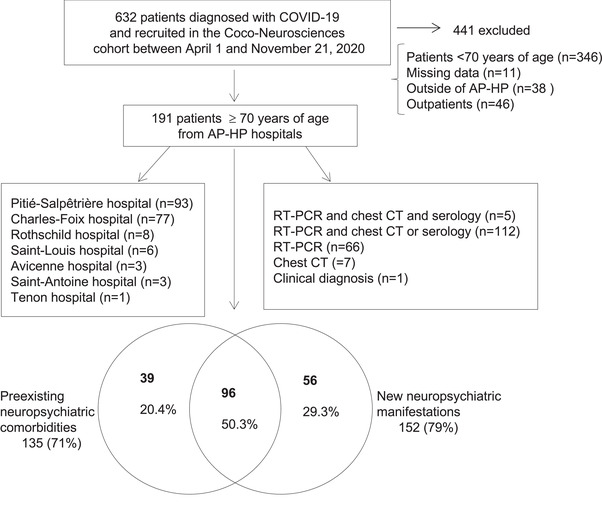
Patient inclusion flowchart. *Abbreviations*: AP‐HP, Assistance Publique‐Hôpitaux de Paris; COVID‐19, coronavirus disease 2019; CT, computed tomography; RT‐PCR, real time‐polymerase chain reaction

This study was part of the “Cohort of Patients with Covid‐19 Presenting Neurological or Psychiatric Disorders” (CoCo‐Neurosciences) (Delorme et al., 2021). COVID‐19 was defined by at least one of the three following criteria: (a) positive severe acute respiratory syndrome coronavirus 2 (SARS‐CoV‐2) real time‐polymerase chain reaction (RT‐PCR) from swab, or positive antibody tests; (b) typical chest computed tomography‐scan (chest CT) findings for SARS‐CoV‐2 infection during the pandemic; (c) suspected COVID‐19 infection according to WHO criteria (2020) (see details of COVID‐19 diagnoses in our patients in Figure 1). We selected patients aged 70 years or older, hospitalized between April 1 and November 21, 2020 in medical wards, mostly neurological or geriatric wards, at Assistance Publique – Hôpitaux de Paris (AP‐HP) hospitals who had (i) COVID‐19 infection, (ii) preexisting neuropsychiatric comorbidities, (iii) and/or new neuropsychiatric manifestations. We excluded patients under 70 years of age, admitted outside of the AP‐HP hospitals, outpatients, and patients with missing age or gender data (Figure 1).

All patients (or their relatives in case of incapacity) received written information about the research and consented to the use of their data. The study received approval from the Sorbonne University Ethics Committee (No. 2020 CER‐202028) and is registered on the clinicaltrials.gov website (NCT04362930).

### 2.2 Data collection

On admission and during hospitalization, treating physicians filled out the CoCo‐Neurosciences standardized electronic data collection including demographic, baseline data, comorbidities, COVID‐19 symptoms, new neurological and psychiatric manifestations, treatments, outcome, and paraclinical findings. Then a group of doctors including neurologists, a geriatrician, and a radiologist reviewed medical records, completed data as well as possible and obtained follow‐up information about survivors after hospital discharge.

### 2.3 Criteria of the studied variables

Autonomy was assessed using the activities of daily living (ADL) (Katz & Akpom, 1976). Frailty was assessed with the adjusted Rockwood clinical frailty scale (Rockwood et al., 2005).

Neurological comorbidities included a history of cognitive impairment, Parkinsonism, cerebrovascular disease, brain tumor, epilepsy, amyotrophic lateral sclerosis, peripheral neuropathy, and others neurological disorders. Brain tumors consisted mostly of glioblastoma and primary central nervous system lymphoma. Parkinsonism included Parkinson's disease and related syndromes. Psychiatric comorbidities included a history of depression‐anxiety, bipolar disorders, and psychosis.

New neuropsychiatric symptoms included anosmia, dysgeusia, myalgia, headache, dizziness, sensory symptoms, motor deficit, movement disorder, behavioral and cognitive disturbance, gait and swallowing impairment, impaired consciousness, and delirium. Delirium was assessed using the Confusion Assessment Method (Inouye et al., 2014). Impaired consciousness was reported when it was unrelated to sedative and hypnotic agents used in intensive care units (ICUs). Psychiatric symptoms included anxiety, mood and sleep disturbances, psychotic signs, symptoms of post‐traumatic stress, and symptoms of catatonia. Atypical manifestations such as falls are common in older patients with COVID‐19 (Annweiler et al., 2021; Karlsson et al., 2021). Falls occurring as an initial manifestation of COVID‐19 or during infection were included.

New‐onset neurological disorders were diagnosed based on a combination of clinical and paraclinical data and included encephalopathy, cerebrovascular disease, epileptic seizures, encephalitis, myelitis, and Guillain‐Barré syndrome. Acute encephalopathy was defined according to Slooter and al. (2020) and was considered toxic‐metabolic in nature if abnormalities were noted on electrolytes, glycemia, uremia, renal/hepatic functions, or blood gases, or on electroencephalogram (EEG), when available. Cerebrovascular disease, including new ischemic stroke, intracerebral hemorrhage, or cerebral venous sinus thrombosis were diagnosed by specialized vascular neurologists. Traumatic intracranial hemorrhages were excluded.

Epileptic seizures included new seizures or exacerbation of seizures in patients with known epilepsy. Diagnoses of acute encephalitis, myelitis, and Guillain‐Barré syndrome were based on the criteria of the Liverpool Brain Infections Group (Ellul et al., 2020). Critical illness polyneuropathy or myopathy unrelated to COVID‐19 was diagnosed in the recovery phase after sedative drug withdrawal in post‐ICU wards by intensive care physicians and neurologists.

Other clinical, treatment, and paraclinical variables are defined in Supporting Information S1. We used the neutrophil to lymphocyte (N/L) ratio to reflect the combination of inflammatory response and immunity imbalance. We built receiver operating characteristic curves for the N/L ratio, and by maximizing the Youden index, we identified the best N/L ratio thresholds to predict mortality risk was ≥ 9.9 with an area under curve at 0.7. We included this cut‐off in analysis.

### 2.4 Outcome

The main outcome was the survival time and mortality from COVID‐19 at 40 days. All deaths occurring within 40 days from first symptoms of COVID‐19 were recorded. Survival time was the number of days from the first symptoms of COVID‐19 until death or the end of the 40 day‐observation period. The 40‐day endpoint was chosen because there was no loss of follow‐up before 40 days, and we believe it is long enough to reflect the mortality risk related to COVID‐19, taking into account both respiratory and neurological complications.

### 2.5 Statistical analysis

Continuous variables are presented as median [interquartile range (IQR)], as they were non‐normally distributed using the Shapiro‐Wilk test. Categorical variables are presented as numbers (percentages). Missing data were not imputed and are described in Supporting Information S2.

The Kaplan‐Meier estimator was used to compute the survival curve.

Cox proportional‐hazards models were used to assess the potential factors of instantaneous risk of mortality (RoM) from COVID‐19 infection. This analysis consisted of three steps: (1) univariate analysis on all variables; (2) forward stepwise analysis including variables from univariate analysis with *p* < .1 and less than 15% missing data in nonsurvivors. Among two paraclinical variables of the same category (white blood cell (WBC) count and WBC groups, neutrophil count and neutrophil groups, platelets and thrombocytopenia, serum creatinine, and glomerular filtration rate), we included the one with smaller *p*. The N/L ratio cut‐off was included rather than continuous N/L ratio value (both with *p* < .001 on univariate analysis) because the clinical application of a cut‐off is more direct. We excluded lymphocyte count because this value was used to calculate the N/L ratio; (3) Multivariate analysis: using the three most significant variables to build a multivariate model to avoid overfitting the model because of a limited number of deaths (*n* = 37). *p‐*Values < .05 were considered significant on multivariate analysis.

The proportional hazards assumptions of the Cox models were checked using the Schoenfeld residuals test. Analyses were performed using STATA^R^ version 16.1 (Stata Corp., College Station, TX, USA).

## 3 RESULTS

### 3.1 Participants, baseline characteristics, and neuropsychiatric conditions

A total of 191 patients aged 70 years or older (median age 80 [IQR 74–87]) were recruited. Of them, 51% were male, 17% were > 90 years of age. The median [IQR] ADL score for autonomy was 5.5 [4–6] and Rockwood clinical scale was 5 [4–6]. Patients had a median of four comorbidities and took six medications daily (Table 1).

**TABLE 1:** Univariate analysis for demographic, baseline characteristics, and comorbidities

Variables	All patients *n* = 191	Survived *n* = 154	Deceased *n* = 37	Hazard ratio (95%, CI)	*p*
Age, years, median [IQR]	80 [74–87]	79.5 [74–87]	82.0 [76–88]	1.05 [0.97–1.05]	.47
Age groups					.51
70–80	97 (50.8%)	81 (52.6%)	16 (43.2%)	Ref.	
81–90	62 (32.5%)	47 (30.5%)	15 (40.5%)	1.52 (0.75–3.10)	.24
91–100	32 (16.7%)	26 (16.9%)	6 (16.2%)	1.17 (0.46–3.00)	.74
Male gender	97 (50.8%)	71 (46.1%)	26 (70.2%)	2.53 (1.25–5.12)	**.007**
Ethnic origin					**.048**
Caucasian	123 (69.9%)	100 (69.4%)	23 (71.9%)	Ref.	
North African	29 (16.5%)	27 (18.7%)	2 (6.3%)	0.32 (0.78–1.39)	.13
Sub‐Saharan African	7 (3.9%)	6 (4.2%)	1 (3.1%)	0.71 (0.09–5.24)	.73
South Asian	9 (5.1%)	4 (2.8%)	5 (15.6%)	3.06 (1.16–9.04)	**.023**
Caribbean	4 (2.3%)	4 (2.8%)	0	NA	
Others	4 (2.3%)	3 (2.1%)	1 (3.1%)	1.22 (0.16–9.04)	.84
Body mass index, kg/m^2^, median [IQR]	23.7 [21–28]	23.6 [21.2–27.6]	24.7 [20.2–27.4]	0.98 (0.92–1.05)	.62
Numbers of comorbidities, median [IQR]	4 [3–7]	4 [3–6]	4 [3–7]	0.97 (0.86–1.08)	.57
Number of medications, median [IQR]	6 [3–8]	6 [3–8]	6 [4–8]	1.03 (0.95–1.12)	.49
Anticoagulant treatment	47 (24.7%)	35 (22.9%)	12 (32.4%)	1.54 (0.78–3.10)	.22
Autonomy					
Living conditions					.35
Own home	118 (61.8%)	97 (63.0%)	21 (56.8%)	Ref.	
Nursing home	18 (9.42%)	12 (7.8%)	6 (16.2%)	2.02 (0.81–5.01)	.28
Hospitalized^a^	55 (28.8%)	45 (29.2%)	10 (27.0%)	1.00 (0.47–2.14)	.98
ADL, median [IQR]	5.5 [4–6]	6 [4–6]	4.8 [1.7–6]	0.78 (0.67–0.92)	**.002**
Frailty Rockwood score, median [IQR]	5 [4–6]	4 [3–6]	5.5 [4–6]	1.28 (1.00–1.65)	**.049**
Comorbidities					
Body mass index ≥ 30 kg/m2	25 (15.7%)	21 (16.3%)	4 (13.3%)	0.83 (0.29–2.38)	.73
Cardiac disease	124 (65.0%)	101 (65.6%)	23 (62.2%)	0.87 (0.44–1.69)	.68
Lung disease	33 (17.3%)	26 (16.9%)	7 (18.9%)	1.19 (0.52–2.72)	.67
Current smoking	23 (12.0%)	20 (12.9%)	3 (8.1%)	0.62 (0.19–2.01)	.42
Diabetes	46 (24.1%)	38 (24.7%)	8 (21.6%)	0.85 (0.39–1.85)	.68
Chronic kidney disease	41 (22.2%)	35 (23.0%)	6 (18.2%)	0.78 (0.32–1.88)	.57
Immunodepression	22 (11.5%)	14 (9.09%)	8 (21.6%)	2.28 (1.04–4.99)	**.04**
Cancer	35 (18.3%)	24 (15.6%)	11 (29.7%)	2.11 (1.04–4.28)	**.04**
Neuropsychiatric comorbidities					
Cognitive impairment	74 (38.7%)	59 (38.3%)	15 (40.5%)	1.10 (0.57–2.13)	.77
Parkinsonism	12 (6.3%)	7 (4.6%)	5 (13.5%)	2.67 (1.03–6.85)	**.04**
Cerebrovascular disease	41 (21.5%)	35 (22.7%)	6 (16.2%)	0.73 (0.30–1.74)	.48
Brain tumor	12 (6.3%)	7 (4.6%)	5 (13.9)	2.81 (1.09–7.25)	**.03**
Epilepsy	6 (3.1%)	6 (3.9%)	0	NA	
Amyotrophic lateral sclerosis	2 (1.1%)	0	2 (5.4%)	NA	
Peripheral neuropathy	4 (2.1%)	4 (2.6%)	0	NA	
Others neurological disorders	5 (2.6%)	5 (3.3%)	0	NA	
Depression‐anxiety	55 (28.8%)	45 (29.2%)	10 (27.0%)	0.96 (0.46–1.98)	.92
Bipolar disorder	4 (2.1%)	4 (2.6%)	0	NA	
Psychosis	5 (2.6%)	5 (3.3%)	0	NA	

*Note*: Values in bold indicate *p* < .1.

Abbreviations: ADL, activities of daily living score; CI, confidence interval; IQR, interquartile range; NA, not applicable; Ref., reference category for the calculation of hazard ratios in categorical variables with more than two categories.

^a^ Already hospitalized for other reason when affected by COVID‐19.

Out of 191 patients, 135 (71%) had preexisting neurological or psychiatric comorbidities, 152 (79%) had new‐onset neurological or psychiatric symptoms/disorders, 96 (50%) had both preexisting and new‐onset neurological or psychiatric conditions (Figure 1). A wide variety of new neuropsychiatric symptom and disorder were observed (Table 2). Among cases of encephalopathy (*n* = 116), toxic‐metabolic encephalopathy was diagnosed in 106 patients based on combination of clinical and laboratory or EEG findings.

**TABLE 2:** Univariate analysis for symptoms, disorders, and treatments

Variables	All patients *n* = 191	Survived *n* = 154	Deceased *n* = 37	Hazard ratio (95%, CI)	*p*
Symptoms					
Fever	132 (69.1%)	110 (71.3%)	22 (59.5%)	0.61 (0.31–1.17)	.14
Cough	111 (58.1%)	94 (61.0%)	17 (45.9%)	0.57 (0.30–1.09)	**.09**
Sputum	27 (14.2%)	19 (12.4%)	8 (21.6%)	1.73 (0.79–3.80)	.17
Rhinorrhea	19 (10.0%)	16 (10.5%)	3 (8.1%)	0.76 (0.23–2.47)	.65
Odynophagia	6 (3.2%)	6 (3.9%)	0	NA	
Chest pain	9 (4.7%)	9 (5.9%)	0	NA	
Dyspnea	109 (57.1%)	78 (50.6%)	31 (83.8%)	4.33 (1.80–10.4)	**.001**
Abdominal pain	15 (7.9%)	8 (5.2%)	7 (18.9%)	3.23 (1.41–7.37)	**.005**
Nausea or vomiting	16 (8.4%)	14 (9.15%)	2 (5.4%)	0.61 (0.15–2.55)	.50
Diarrhea	30 (15.8%)	27 (17.6%)	3 (8.1%)	0.43 (0.13–1.40)	.16
Anorexia	50 (26.3%)	41 (26.8%)	9 (24.3%)	0.88 (0.42–1.86)	.74
Fatigue	110 (57.6%)	85 (55.2%)	25 (67.6%)	1.63 (0.82–3.24)	.17
Arthralgia	2 (1.1%)	1 (0.6%)	1 (2.8%)	3.02 (0.41–22.05)	.28
Skin lesions	24 (12.6%)	19 (12.4%)	5 (13.5%)	1.09 (0.42–2.81)	.85
Fall	33 (17.6%)	24 (15.7%)	9 (25.7%)	1.72 (0.81–3.67)	.16
Neurological symptoms					
Anosmia	12 (6.3%)	10 (6.5%)	2 (5.4%)	0.82 (0.20–3.40)	.79
Ageusia	8 (4.2%)	7 (4.5%)	1 (2.7%)	0.60 (0.08–4.40)	.62
Myalgia	19 (9.9%)	13 (8.4%)	6 (16.2%)	1.76 (0.74–4.22)	.20
Headache	11 (5.8%)	9 (5.8%)	2 (5.4%)	0.88 (0.21–3.66)	.86
Dizziness	4 (2.1%)	4 (2.6%)	0	NA	
Sensory symptoms	11 (5.9%)	9 (6.0%)	2 (5.4%)	0.96 (0.23–3.99)	.96
Motor deficit	21 (11.4%)	18 (12.0%)	3 (8.6%)	0.73 (0.22–2.38)	.60
Movement disorder	2 (1.1%)	2 (1.3%)	0	NA	NA
Behavioral disturbance	33 (17.7%)	27 (18.0%)	6 (16.7%)	0.91 (0.38–2.20)	.84
Cognitive disturbance	44 (23.0%)	38 (24.7%)	6 (16.2%)	0.61 (0.26–1.45)	.26
Gait impairment	20 (10.7%)	15 (10%)	5 (13.9%)	1.43 (0.45–3.68)	.46
Swallowing impairment	15 (7.9%)	12 (7.8%)	3 (8.1%)	1.04 (0.32–3.40)	.94
Impaired consciousness	34 (17.8%)	23 (14.9%)	11 (29.7%)	2.10 (1.04–4.26)	**.04**
Delirium	93 (48.7%)	71 (46.1%)	22 (59.5%)	1.64 (0.85–3.17)	.14
Psychiatric symptoms					
Depression‐Anxiety	35 (18.7%)	32 (21.2%)	3 (8.3%)	0.38 (1.12–1.23)	.11
Psychotic signs	11 (5.9%)	8 (5.3%)	3 (8.3%)	1.53 (0.47–5.00)	.48
Post‐traumatic stress	NA	3 (2.0%)	NA	NA	
Sleep disturbance	16 (8.4%)	13 (8.5%)	3 (8.1%)	0.96 (0.29–3.13)	.95
Catatonia	2 (1.1%)	2 (1.3%)	0	NA	NA
Neurological disorders					
Encephalopathy	116 (60.7%)	89 (57.8%)	27 (72.9%)	1.85 (0.89–3.82)	**.09**
Cerebrovascular disease	11 (5.8%)	8 (5.2%)	3 (8.3%)	1.53 (0.47–4.99)	.48
Encephalitis	6 (3.2%)	5 (3.3%)	1 (2.8%)	0.80 (0.11–5.82)	.82
Seizures	8 (4.2%)	5 (3.3%)	3 (8.3%)	2.16(0.66–7.04)	.20
Guillain Barré syndrome	1 (0.5%)	1 (0.6%)	0	NA	NA
Myelitis	0	NA	NA	NA	NA
Treatments					
Hydroxychloroquine	23 (12.0%)	20 (12.9%)	3 (8.1%)	0.63 (0.19–2.02)	.43
Antiviral treatments	17 (8.9%)	15 (9.7%)	2 (5.4%)	0.57 (0.14–2.38)	.44
Corticosteroids	42 (22.1%)	26 (17.0%)	16 (44.4%)	4.04 (1.57–5.88)	**.001**
Immunomodulatory/AC agents	14 (7.3%)	12 (7.8%)	2 (5.4%)	0.66 (0.16–2.75)	.56
Antibiotics	142 (74.3%)	112 (72.3%)	30 (81.1%)	1.51 (0.66–3.43)	.33
Oxygen‐therapy	139 (72.8%)	104 (67.5%)	35 (94.6%)	7.40 (1.78–30.7)	**.006**
Median flow, L/min max 15, median [IQR]	4 [2–12]	4 [2–10]	12 [4–15]	1.10 (1.03–1.18)	**.004**
Flow at 15 L/min	27 (22.5%)	14 (15.4%)	13 (44.8%)	3.14 (1.50–6.54)	**.002**
Treatment for AHRF	51 (26.7%)	34 (22.1%)	17 (45.9%)	2.50 (1.31–4.78)	**.005**
Admission in intensive care unit	29 (14.7%)	24 (15.6%)	5 (13.5%)	0.84 (0.32–2.14)	.71
Critical illness neuromyopathy	13/22	13/22	NA	NA	NA

*Note*: Values in bold indicate *p* < .1.

Abbreviations: AC: anticytokine agents; AHRF, acute hypoxemic respiratory failure; CI, confidence interval; IQR, interquartile range; NA, not applicable; Ref., reference category for the calculation of hazard ratios in categorical variables with more than two categories.

### 3.2 Mortality

**FIGURE 2:**
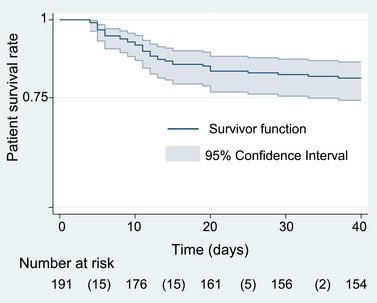
The Kaplan‐Meier survival estimate for the 191 older patients with COVID‐19 and neuropsychiatric conditions. *Note*: Number of deaths in parentheses

At 40 days after the onset of COVID‐19 symptoms, 37 of 191 (19.4%) of patients had died following a median [IQR] of 11 days [6–14] (Figure 2). The median follow‐up [IQR] for survivors was 167 days [106–241] as of January 2021.

### 3.3 Univariate analysis for risk factors of mortality

The results of univariate analysis were presented in Tables 1, 2, 3 and Table S1. Variables with *p* < .1 and less than 15% of missing data in nonsurvivors were candidates for the stepwise analysis.

**TABLE 3:** Univariate analysis for routine blood test findings

Variables	All patients *n* = 191	Survived *n* = 154	Deceased *n* = 37	Hazard ratio (95%, CI)	*p*
Hb, g/dL, median [IQR]	11.8 [10.4–13.3]	11.8 [10.5–13.3]	12.0 [9.9–14.0]	1.02 (0.86–1.20)	.79
Anemia	113 (59.8%)	92 (59.7%)	21 (60.0%)	0.96 (0.49–1.89)	.91
WBC count, × 10^9^/L, median [IQR]	7.3 [5.3–10.4]	7.0 [4.9–10.1]	9.1 [6.5–12.5]	1.08 (1.03–1.13)	**.001**
WBC groups					**.09**
<4	26 (13.8%)	24 (15.6%)	2 (5.9%)	0.44 (0.10–1.91)	.28
4–10	108 (57.5%)	90 (58.4%)	18 (52.9%)	Ref	
>10	54 (28.7%)	40 (26.0%)	14 (41.2%)	1.67 (0.83–3.38)	.15
Neutrophil, × 10^9^/L, median [IQR]	5.8 [3.6–8.0]	5.5 [3.3–7.6]	7.8 [5.2–9.4]	1.11 (1.04–1.19)	**.003**
Neutrophil groups					**.03**
<1.7	15 (8.1%)	13 (8.5%)	2 (6.1%)	1.13 (0.25–5.07)	.87
1.7–7.0	101 (54.6%)	89 (58.6%)	12 (36.4%)	Ref.	
>7.0	69 (37.3%)	50 (32.9%)	19 (57.6%)	2.52 (1.22–5.19)	**.01**
Lymphocyte, × 10^9^/L, median [IQR]	0.77 [0.55–1.07]	0.82 [0.60–1.09]	0.57 [0.35–0.81]	0.19 (0.06–0.57)	**.003**
Lymphopenia < 1.5	173 (92.1%)	140 (91.5%)	33 (97.1%)	2.91 (0.39–21.3)	.29
N/L ratio, median [IQR]	6.1 [3.6–11.2]	5.9 [3.6–9.6]	11.3 [5.8–18.6]	1.05 (1.03–1.07)	**<.001**
Cut‐off ≥ 9.9	60 (32.6%)	38 (24.8%)	22 (66.7%)	4.83 (2.34–9.98)	**<.001**
Platelets, × 10^9^/L, median [IQR]	223 [163–291]	233 [170–293]	192 [121–258]	0.99 (0.99–1.00)	**.006**
Thrombocytopenia < 150	33 (17.6%)	17 (11.1%)	16 (47.1%)	5.34 (2.72–10.50)	**<.001**
Albumin, g/L, median [IQR]	29 [26–32.6]	29 [26–33]	29 [27–33]	1.02 (0.95–1.10)	.49
Calcium adjusted, mmol/L, median [IQR]	2.46 [2.36–2.57]	2.48 [2.37–2.58]	2.38 [2.24–2.50]	0.19 (0.03–1.01	**.052**
Glycemia, mmol/L, median [IQR]	5.9 [5.2–7.3]	5.8 [5.2–7.2]	6.8 [5.6–8.1]	1.20 (1.05–1.36)	**.006**
Sodium, mmol/L, median [IQR]	139 [136–141]	138 [136–141]	140 [137–144]	1.05 (1.00–1.10)	**.015**
Potassium, mmol/L, median [IQR]	4 [3.6–4.3]	4 [3.6–4.3]	4 [3.8–4.4]	1.30 (0.66–2.55)	.44
Uremia, mmol/L, median [IQR]	8.3 [5.7–11.3]	8 [5.3–10.5]	10 [8–17]	1.06 (1.03–1.09)	**<.001**
Osmolarity, mOsm, median [IQR]	298 [292–307]	298 [292–306]	304 [300–324]	1.03 (1.01–1.04)	**<.001**
Creatinine, μmol/L, median [IQR]	85 [67–108]	82 [66–105]	96 [74–132]	1.00 (1.00–1.00)	**.018**
Glomerular filtration rate, ml/min/1,73 m^2^, median [IQR]	70.6 [47.8–83.5]	70.9 [51.4–83.6]	59.5 [39.5–81.4]	0.99 (0.97–1.00)	**.074**
C‐reactive protein, mg/L, median [IQR]	95 [55–170]	95 [49–158]	141 [68–177]	1.00 (0.99–1.00)	.146
ASAT, U/L, median [IQR]	39 [27–61]	37 [27–56]	44 [30–106]	1.005 (1.00–1.01)	**<.001**
ALAT, U/L, median [IQR]	28 [18–46]	28 [19–46]	31 [18–47]	1.004 (0.99–1.01)	.21

*Note*: Values in bold indicate *p‐v*alue < .1.

Abbreviations: ALAT, alanine aminotransferase; ASAT, aspartate aminotransferase; CI, confidence interval; Hb, hemoglobin; IQR, interquartile range; N/L, neutrophil/lymphocyte; Ref., reference category for the calculation of hazard ratios in categorical variables with more than 2 categories; WBC, white blood cell.

Preexisting factors such as male gender, South Asian ethnicity as compared to Caucasian ethnicity, a lower ADL score, a higher Rockwood score, comorbidities including immunodepression, cancer, brain tumors, and Parkinsonism, new symptoms including dyspnea and abdominal pain, impaired consciousness, treatments including corticosteroids, oxygen therapy, high‐flow oxygen therapy at 15 L/min, and treatment for acute hypoxemic respiratory failure were associated with a higher RoM (all *p* < .05) (Tables 1 and 2). Among 29 patients admitted to the ICUs, 13 of 22 patients with mechanical ventilation presented critical illness neuromyopathy, and all 13 were still alive at 40 days (median follow‐up [IQR] 186 days [148–238]).

Elevated WBC and neutrophil counts, decreased lymphocyte and platelet counts, the presence of neutrophilia and thrombocytopenia, higher N/L ratio, a N/L ratio cut‐off of ≥ 9.9, higher levels of glycemia, sodium, uremia, osmolarity, creatinine, aspartate aminotransferase, serum ferritin, lower level of prothrombin time (in percentage) were associated with a higher RoM (all *p* < .05) (Table 3, Table S1).

### 3.4 Multivariate analysis for risk factors of mortality

Among variables included in forward stepwise analysis that remained significant (*p* < .05) (Table 4), the three most significant were included in a final multivariate analysis and were independently associated with a higher RoM (all *p* < .001): lower ADL scores (HR = 0.69, 95% CI: 0.58–0.82), a N/L ratio ≥ 9.9 (HR = 5.69, 95% CI: 2.69–12.0) and thrombocytopenia (HR = 5.70, 95% CI: 2.75–11.8).

**TABLE 4:** Multivariate analysis for risk of mortality at 40 days after forward stepwise selection

		Final multivariate model	
Variables included in forward stepwise	*p‐*Values from forward stepwise	Hazard ratio (95% CI)	*p*
Male gender	**.016**		
South Asian ethnicity	**.002**		
Activities of daily living score	**<.001**	0.69 (0.58–0.82)	**<.001**
Frailty Rockwood score	>.05		
Immunodepression	>.05		
Cancer	>.05		
Brain tumor	**.012**		
Parkinsonism	>.05		
Dyspnea	**.001**		
Cough	>.05		
Abdominal pain	**.002**		
Impaired consciousness	**.002**		
Encephalopathy	>.05		
Corticosteroids	>.05		
Oxygen therapy	>.05		
Treatment for AHRF	>.05		
White blood cell count, × 10^9^/L	>.05		
Neutrophil, × 10^9^/L	>.05		
Neutrophil to lymphocyte ratio ≥ 9.9	**.001**	5.69 (2.69−12.0)	**<.001**
Thrombocytopenia < 150 × 10^9^/L	**<.001**	5.70 (2.75–11.8)	**<.001**
Sodium, mmol/L	>.05		
Uremia, mmol/L	>.05		
Osmolarity, mOsm	>.05		
Creatinine, μmol/L	>.05		

*Note*: Values in bold indicate *p* < .05.

Abbreviations: AHRF: acute hypoxemic respiratory failure; CI: confidence interval.

The test of proportional hazards assumption validated the model (*p* = .38) with a total number of patients of 183/191 used in the model.

## 4 DISCUSSION

To the best of our knowledge, this is the first study to examine the risk factors for mortality in older inpatients with COVID‐19 and neuropsychiatric conditions, taking into account preexisting and new‐onset neuropsychiatric conditions, together with general and paraclinical findings. We found a mortality rate of 19.4% at 40 days. Among several factors found on univariate analysis, male sex, a history of brain tumor or Parkinsonism or the occurrence of impaired consciousness were associated with a higher RoM. On multivariate analysis, lower ADL scores for autonomy, an N/L ratio ≥ 9.9, and thrombocytopenia were independently associated with a higher RoM.

### 4.1 Mortality rates

The mortality rate of our patents (19.4%) appears higher than in patients under the age of 70 (between 0% and 18.7%) (Richardson et al., 2020) but lower than that reported in other geriatric cohorts (between 24% and 47%) (Covino et al., 2021; Hägg et al., 2020; Ramos‐Rincon et al., 2021; Zerah et al., 2021). In comparison to a large French cohort of patients ≥ 70 years of age from acute geriatric wards, our population appeared younger, had higher ADL scores for autonomy, with only 9% of patients who were initially in nursing homes, compared to 29% (Zerah et al., 2021). Older patients in nursing homes with advanced neurological comorbidities, in particular dementia, and severe COVID‐19 may not have been transferred to hospitals. Moreover, our recruitment may have excluded patients with severe non‐neurological complications of COVID‐19.

### 4.2 Mortality risk factors related to preexisting conditions, baseline functional status, and neurological comorbidities

Unlike studies in patients of all ages (Docherty et al., 2020) or aged 18 years or older (Cummings et al., 2020), studies in older patients often did not show old age (Hägg et al., 2020; Steinmeyer et al., 2020; Zerah et al., 2021), or major comorbidities including chronic cardiac and respiratory disease, as risk factors for mortality (Hägg et al., 2020; Ramos‐Rincon et al., 2021; Steinmeyer et al., 2020; Zerah et al., 2021) as older populations are often more homogeneous. However, male sex was associated with higher mortality in adult cohorts (Docherty et al., 2020; Richardson et al., 2020) and remained significant in many studies in older patients (Mendes et al., 2020; Ramos‐Rincon et al., 2021; Zerah et al., 2021) as well as in our study.

A lower ADL score was independently associated with a higher RoM, confirming previous studies (Covino et al., 2021; Zerah et al., 2021). Reduced autonomy increases vulnerability in these older patients, and nongeriatric physicians should systematically adopt this score in routine, as do geriatricians, to be aware of their higher risk for mortality in order to adapt treatment and better explain prognosis to loved ones.

Globally, data are sparse for patients with brain tumors and COVID‐19. A cohort of 87 patients under the age of 25 with cancer, including three patients with brain tumors showed no death related to COVID (Parker et al., 2022). Certain cohorts of older patients have found an association between cancer/malignity and death (Covino et al., 2021) while others have not (Genet et al., 2020; Li et al., 2020; Neumann‐Podczaska et al., 2020; Ramos‐Rincon et al., 2021; Vrillon et al., 2020; L. Wang et al., 2020; Zerah et al., 2021), but these studies did not mention brain tumors (Covino et al., 2021; Genet et al., 2020; Li et al., 2020; Neumann‐Podczaska et al., 2020; Ramos‐Rincon et al., 2021; Vrillon et al., 2020; L. Wang et al., 2020; Zerah et al., 2021). In our study, the presence of a brain tumor was associated with a higher RoM (*p* = .03), and this association remained significant (*p* = .012) after stepwise analysis. Brain tumors were active medical conditions in our patients who were often undergoing chemotherapy, which could contribute to their overall fragile condition and contribute to poor outcomes.

Parkinsonism‐related pathology may lead to the rigidity of certain respiratory muscles which may impair the cough reflex, therefore contributing to poor prognosis in patients with SARS‐CoV‐2. Parkinson's disease patients have a higher risk of SARS‐CoV‐2 (Yu et al., 2021). Meta‐analyses including a cohort of younger patients (mean age 58 years) (Zhang et al., 2020) and older patients in nursing homes (Rutten et al., 2020) (mean age 84 years) showed an association between Parkinson's disease and mortality from COVID‐19 (Putri et al., 2021), but this association was not found in inpatient geriatric cohort (Zerah et al., 2021). Our study showed an association (*p* = .04) between Parkinsonism and a higher RoM, but this association was not independent. Patients with advanced Parkinson's disease appear to be particularly vulnerable (Fearon, 2021). Further studies with large cohorts of geriatric patients with COVID‐19 and advanced Parkinson's disease are needed to provide more information.

Cognitive impairment is a common condition in older patients. Several studies have reported an association between dementia and mortality in patients with COVID‐19 (Hariyanto et al., 2021; N. Liu et al., 2020), while our study and several others did not (De Smet et al., 2020; Genet et al., 2020; Graham et al., 2020; Mendes et al., 2020; Ramos‐Rincon et al., 2021; Steinmeyer et al., 2020; Zerah et al., 2021). This absence of association is probably due to a more homogeneous study population. Indeed, the studies that did not show an association (De Smet et al., 2020; Genet et al., 2020; Graham et al., 2020; Mendes et al., 2020; Ramos‐Rincon et al., 2021; Steinmeyer et al., 2020; Zerah et al., 2021) consisted of very old patients (average age between 80 and 87 years), with a high prevalence of dementia (ranging from 30% to 88%). Moreover, our cohort of older patients with neuropsychiatric conditions may have had more aggressive neurological conditions than dementia, conditions that may be associated with an even higher risk of mortality.

### 4.3 Mortality risk factors related to new‐onset neurological symptoms or disorders during the course of COVID‐19 infection

A study of adult patients aged 18 years or older (mean age 63) showed that coma predicted death in patients hospitalized for COVID, independent of age (Boehme et al., 2022). In studies with older patients, impaired consciousness has often been integrated into a score (Covino et al., 2021; Zerah et al., 2021), but rarely examined as an individual symptom (Annweiler et al., 2021; Sun et al., 2020), or in association with mortality (Sun et al., 2020). We found that impaired consciousness was associated with a higher RoM, and this association remained significant after stepwise analysis (*p* = .002). Impaired consciousness is often caused by severe brain damage or serious non‐neurological conditions including respiratory failure, or even multiorgan dysfunction which may explain our findings.

Encephalopathy was shown to be associated with a higher risk of mortality in a mixed (outpatients and inpatients) cohort of adult patients aged 18 years or older with COVID‐19 and de novo neuropsychiatric manifestations (Delorme et al., 2021) and tended towards an association with a higher RoM (*p* < .1) in our study. The high prevalence (61%) of encephalopathy in our older patients explains this weak association. We found no corresponding study in older patients, but encephalopathy was the most frequent (20%) neurological complication in older patients who died from COVID‐19 (Martín‐Jiménez et al., 2020). A toxic‐metabolic origin was suggested in most cases with encephalopathy in our study. Correction of toxic‐metabolic perturbations could improve outcome for these patients.

Delirium is a common complication of COVID‐19 in older patients and has been shown to be associated with an increased risk of mortality (Shao et al., 2021). Studies showing no association between delirium and mortality (Vrillon et al., 2020, 2021, our study) seemed to be more homogeneous with a higher prevalence of delirium (48–82%) as compared to the prevalence found in a metanalysis (24% for all patients and 28% for patients aged over 65 years) (Shao et al., 2021).

### 4.4 Mortality risk factors related to biological markers during the course of COVID‐19 infection

An N/L ratio ≥ 9.9 strongly and independently predicted RoM in our study. Its robust value in predicting COVID‐19 severity and mortality has been demonstrated in adult and younger patients (aged 18 years or older; Liao et al., 2020), median age 48 years; S. Wang et al., 2021, mean age 53; Y. Liu et al., 2020) with interestingly, a similar cut‐off value for the N/L ratio (> 9.13) (Liao et al., 2020). A high N/L ratio may reflect an increased inflammatory response, an immune imbalance, or a combination of the two. The ratio can remain significant in predicting mortality risk in inpatients with COVID‐19 even when neutrophil or lymphocyte counts alone do not (Y. Liu et al., 2020; S. Wang et al., 2021). However, this ratio has rarely been studied in older people. One study included the N/L ratio into a 10‐item score which had a good predictive value for in‐hospital death in older adults aged 60 years or older (Covino et al., 2021). Another study in patients aged 70 years or older showed an increase in the likelihood of death in patients with a higher N/L ratio, but this study included few symptoms of COVID, no neurological symptoms and only two biological variables (Knopp et al., 2020). Here, we showed that the N/L ratio ≥ 9.9 predicted an increased RoM, independently of several other neurological and non‐neurological variables. This variable is particularly simple to obtain because any entry blood workup includes a blood cell count, and the ratio is rapid to calculate.

Thrombocytopenia was also found to be an important independent mortality risk factor in our study. In addition, a lower prothrombin time (expressed in percentage) was associated with a higher RoM on univariate analysis (*p* = .04). These findings echo the results of a study in an adult population of 18 years or older (Liao et al., 2020), where a similar coagulopathy profile was associated with mortality. Previous studies in older patients did not show an association between platelet count and mortality (Neumann‐Podczaska et al., 2020; Steinmeyer et al., 2020; Vrillon et al., 2020, 2021; Zerah et al., 2021), but one study showed a significant decrease in platelets in nonsurvivors (L. Wang et al., 2020). Coagulation laboratory markers vary greatly over the course of COVID‐19 and platelet count decreased in patients with progressively severe illness (Liao et al., 2020), which may explain these conflicting results as markers may have been tested at different points during the disease. It is unclear which mechanisms lead to thrombocytopenia in COVID‐19, but thrombocytopenia is common in viral infections, possibly due to immunological platelet destruction, inappropriate platelet activation and consumption, and impaired megakaryopoiesis (Amgalan & Othman et al., 2020).

We found that lymphocyte count was associated with a higher RoM, but the reference value for lymphopenia < 1.5 × 10^9^/L (92% of our patients) was not. A lower threshold of lymphopenia < 0.8 × 10^9^/L significantly predicted mortality in older patients (Ramos‐Rincon et al., 2021; Steinmeyer et al., 2020). Moreover, the median lymphocyte count value was very low in nonsurvivors between 0.51× 10^9^/L and 0.57 × 10^9^/L in our study and in other older populations (Sun et al., 2020; L. Wang et al., 2020). Studies in younger patients (mean age 62–68 years) (Yan et al., 2020) showed that a low lymphocyte count was associated with death, and the value in nonsurvivors was very low (0.33 × 10^9^/L) as compared to survivors (0.97 × 10^9^/L). We suggest that when evaluating the severity and risk of mortality in patients with COVID‐19, clinicians should consider a lower lymphocyte count threshold.

### 4.5 Limitations

Our population does not represent a general geriatric population, but our findings will be useful for older patients seen in neurological, psychiatric or specific geriatric settings. Geriatricians and neurologists often encounter in real‐life conditions older patients with both new symptoms and significant medical and neurological histories. Our findings therefore can be applied to a real‐world setting and aid neurologists treating older patients and geriatricians who manage patients with neurological disease. The limited sample size and missing data did not allow for inclusion of all possible variables into a multivariate analysis. Data were retrospectively collected which can lead to bias.

## 5 CONCLUSION

In this study, we identified risk factors for mortality in older inpatients with COVID‐19 and neuropsychiatric conditions. In addition, we reported a wide spectrum of new neuropsychiatric manifestations that can occur during COVID‐19 in older patients. These findings may be helpful for neurologists who manage older patients and geriatricians who treat patients with neuropsychiatric conditions.

## CONFLICT OF INTEREST

Cécile Delorme received a research grant from the FIA for the support of this work, travel funding from Merz Pharma, Boston Scientific and Medtronic, outside of this work. Vincent Navarro served as board member for UCB pharma, LivaNova, GW pharma et EISAI, outside of this work. Jean‐Christophe Corvol received a grant from the Fondation de France (#00113315) for the support of this study, and served on the scientific advisory boards for Biogen, UCB, Prevail Therapeutic, Idorsia, Ever Pharma, Denali, and has received grants from the Michael J Fox Foundation and Sanofi outside of this work. Bertrand Degos has received research support from Orkyn, Elivie, Merz, honoraria for speeches from Ipsen, and travail grant from Orkyn outside of this work. The other authors report no disclosures.

### PEER REVIEW

The peer review history for this article is available at https://publons.com/publon/10.1002/brb3.2787

## Supporting information

Method S1: Criteria of other studied clinical, treatment and paraclinical variables
Method S2: Details on Missing Data
Table S1: Univariate analysis for extended blood test, blood gas, and chest CT findings And Acknowledgement list

## Data Availability

The data that support the findings of this study are available from the corresponding author upon reasonable request.
