# Supplementary material for: Older patients with COVID‐19 and neuropsychiatric conditions: A study of risk factors for mortality
Source: Brain Behav. 2022 Nov 10;12(12):e2787. doi: 10.1002/brb3.2787 (PMC9759137; doi:10.1002/brb3.2787)
Supplement: Supplementary file 1 — Method S1: Criteria of other studied clinical, treatment and paraclinical variables Method S2: Details on Missing Data Table S1: Univariate analysis for extended blood test, blood gas, and chest CT findings And Acknowledgement list [file BRB3-12-0-s001.docx]

**SUPPORTING INFORMATION** (including **4 documents**)

**Method S1**: Criteria of other studied clinical, treatment and paraclinical variables

**Method S2**: Details on Missing Data

**Table S1**: Univariate analysis for extended blood test, blood gas, and chest CT findings

**Acknowledgement list**

**Method S1:**

Criteria of other studied clinical, treatment and paraclinical variables

| **Comorbidities**  Patients considered to be immunocompromised had solid or hematological malignancy with current chemotherapy, were organ transplant recipients or were known to have an HIV infection or were treated with long-term corticosteroids.  Cancers were considered comorbidities if disease was progressive. Cancers in remission were not taken into account.  **Treatments**  Treatment for acute hypoxemic respiratory failure included high-level supplemental oxygen (at a flow rate of 15 L per min or greater) or mechanical ventilation (invasive or noninvasive) [1].  Antiviral, immunomodulatory or anticytokine agents were generally administered through enrollment in clinical trials or were used in patients with severe hyperinflammatory markers at the discretion of treating clinicians in collaboration with infectious disease consultants (compassionate use).  **Paraclinical variables**  Routine blood tests included complete blood cell count, glycemia, urea, electrolytes, C-reactive protein (CRP), renal and hepatic function tests. Values of serum sodium, potassium, urea, and glycemia were used to calculate plasma osmolarity (Serum osmolality (mosm/L) = [(Na + K) x 2] + [urea (mmol/L)] + [glucose (mmol/L)]. Values of serum calcium and albumin were used to calculate calcium adjusted (Serum calcium adjusted (mmol/L) = Calcium + 0.025 (40- Albumin). The CKD-EPI equation was used for calculating the glomerular filtration rate  Extended blood tests included additional inflammatory biomarkers (serum ferritin, fibrinogen, procalcitonin, interleukine-6), coagulation profile (prothrombin time in percentage), creatine kinase, lactate dehydrogenase, markers of thrombosis (D-Dimer) and of acute cardiac decompensation (NT Pro-BNP), high- sensitivity troponin T.  Patients admitted at an early stage of COVID-19-infection may have normal routine biological findings at admission, which can worsen as the disease progresses. To obtain the maximal inflammatory profile, we chose the highest CRP value and the corresponding white blood cell count, and to depict the maximal effect of viral infection on immune blood cells [6], we reported the nadir value of lymphocyte count. We then calculated the neutrophil to lymphocyte ratio (N/L ratio) [49,50] using the nadir value of lymphocyte and the corresponding neutrophil count on the same complete blood count in order to reflect the combination of inflammatory response and immunity imbalance. Others biological results were collected during the same period, as closely as possible to peak CRP.  Chest CT findings included the presence or absence of typical COVID-19 abnormalities, percentage of lung involvement (0-10%, 10-25%, >25%) and features (condensation, ground-glass, crazy-paving, embolic complications) according to the radiology report. |
| --- |

**Method S2**: Details on Missing Data

| Missing values are presented as total number and (number in the survivor group; number in the non-survivor group):  Ethnic origin 15 (10;5)  Body Mass Index 33 (25;8)  Number of Medication 2 (1;1)  Anticoagulant treatment 1 (1;0)  Activity Daily Living score 2 (1;1)  Frailty Rockwood score 6 (3;3)  Chronic kidney disease 6 (2;4)  Brain tumors 2 (1;1)  Sputum 1 (1;0)  Rhinorrhea 1 (1;0)  Odynophagia 1 (1;0)  Chest pain 1 (1;0)  Abdominal pain 1 (1;0)  Nausea or vomiting 1 (1;0)  Diarrhea 1 (1;0)  Anorexia 1 (1;0)  Arthralgia 2 (1;1)  Skin lesion 1 (1;0)  Falls 3 (1;2)  Sensory symptoms 5 (5;0)  Motor deficit 6 (4;2)  Movement disorder 5 (4;1)  Behavioral disturbance 5 (4;1)  Gait impairment 5 (4;1)  Depression-Anxiety 4 (3;1)  Psychotic signs 4 (3;1)  Sleep disturbance 1 (1;0)  Cerebrovascular disease 1 (0;1)  Encephalitis 1 (0;1)  Seizures 1 (0;1)  Corticosteroids 1 (0;1)  Median flow oxygen therapy 20 (14;6)  Flow at 15L/min 19 (13;6)  Hemoglobin 2 (0;2)  White blood cell count 3 (0;3)  Lymphocyte 4 (1;3)  Neutrophil/Lymphocyte ratio 5 (1;4)  Platelets 4 (1;3)  Albumin 12 (3;9)  Calcium adjusted 15 (8;7)  Glycemia 24 (16;8)  Sodium 3 (0;3)  Potassium 3 (0;3)  Uremia 3 (0;3)  Osmolarity 8 (3;5)  Creatinine 3 (0;3)  C-reactive protein 4 (1;3)  Procalcitonin 41 (33;8)  Serum ferritin 31 (20;11)  Interleukine-6 143 (110;33)  Activated fibrinogen 28 (20;8)  Prothrombin time 18 (12;6)  Lactate dehydrogenase 31 (20;11)  Creatine kinase 52 (38;14)  Aspartate aminotransferase 8 (2;6)  Alanine aminotransferase 8 (2;6)  NT Pro-BNP 74 (59,15)  Hs cardiac Troponin T 56 (42;14)  D-Dimer 94 (72;22)  Blood gas 79 (67;12)  Chest CT 30 (21;9) |
| --- |

**Table S1**: Univariate analysis for extended blood test, blood gas, and chest CT findings

| Variables | All patients | Survived | Deceased | Hazard ratio  (95%, CI) | p |
| --- | --- | --- | --- | --- | --- |
|  | n=191 | n=154 | n=37 |  |  |
| **Blood tests** |  |  |  |  |  |
| Procalcitonin, ng/mL,  median [IQR] | 0.21 [0.1-0.56] | 0.19 [0.10-0.46] | 0.38 [0.16-0.92] | 0.98 (0.85-1.13) | 0.78 |
| Serum ferritin, ng/mL,  median [IQR] | 664 [386-1228] | 655 [385-1139] | 849 [468-1665] | 1.00 (1.00-1.00) | **0.014** |
| Interleukine-6, pg/mL,  median [IQR] | 38.8 [16.4-85.4] | 38.8 [14.4-85.5] | 42.1 [31-135] | 0.99 (0.99-1.00) | 0.96 |
| Activated fibrinogen,  g/L, median [IQR] | 5.7 [4.8-7.0) | 5.8 [4.8-7.0] | 5.6 [4.2-6.9] | 0.84 (0.66-1.07) | 0.16 |
| Prothrombin time (%),  median [IQR] | 87 [75-97] | 88 [77-98] | 82 [65-95] | 0.98 (0.97-0.99) | **0.04** |
| LDH, U/L, median [IQR] | 371 [283-477] | 362 [281-457] | 414 [328-531] | 1.00 (0.99-1.00) | **0.09** |
| Creatine kinase, U/L  median [IQR] | 94 [48-329] | 87 [46-290] | 161 [91-571] | 1.00 (0.99-1.00) | 0.41 |
| NTPro-BNP, ng/L,  median [IQR] | 1093 [310-3998] | 791 [302-3022] | 3326 [1099-7237] | 1.00 (0.99-1.00) | 0.42 |
| Hsc troponin T, ng/L,  median [IQR] | 26.9 [16-55] | 25.8 [16-47] | 42 [22-68] | 1.00 (0.99-1.00) | 0.46 |
| D-Dimer, ng/ml,  median [IQR] | 1590 [911-3545] | 1547 [807-3289] | 2680 [1299-8150] | 1.00 (0.99-1.000) | 0.82 |
| **Arterial Blood gas** | | | | | |
| PaO2, mmHg,  median [IQR] | 74 [65-84] | 75 [68-83] | 66 [60-84] | 0.97 (0.95-1.00) | 0.**055** |
| PaCO2, mmHg,  median [IQR] | 35 [32.-40] | 35 [32-40] | 34 [30-41] | 0.99 (0.94-1.04) | 0.88 |
| pH, median [IQR] | 7.46 [7.46-7.5] | 7.46 [7.43-7.40] | 7.45 [7.41-7.49] | 0.03 (0.00-4.55) | 0.17 |
| **Chest CT** | 124 (77%) | 103 (77.4%) | 21 (75%) | 0.88 (0.37-2.06) | 0.77 |
| Lung involvement |  |  |  |  | 0.52 |
| 0-10% | 57 (36.7%) | 49 (38.3%) | 9 (33.3%) | Ref. |  |
| 10-25% | 44 (28.4%) | 34 (26.6%) | 10 (37.0%) | 1.53 (0.62-3.75) | 0.36 |
| >25%  Lesion feature | 54 (34.8%) | 46 (35.9%) | 8 (29.6%) | 0.91 (0.35-2.36) | 0.84 |
| Consolidation | 97 (61.8%) | 80 (61.5%) | 17 (62.9%) | 1.04 (0.47-2.26) | 0.92 |
| Ground-glass opacity | 117 (74.0%) | 100 (76.3%) | 17 (62.9%) | 0.55 (0.25-1.20) | 0.13 |
| Crazy paving | 57 (39.6%) | 46 (38.3%) | 11 (45.8%) | 1.33 (0.60-2.97) | 0.48 |
| Embolic lesion | 15 (9.3%) | 12 (9.0%) | 3 (10.3%) | 1.04 (0.32-3.45) | 0.94 |

In bold: p*-v*alue < 0.1

Abbreviations: IQR, interquartile range; CI, confidence interval; LDH, Lactate dehydrogenase; NTPro-BNP, N-terminal pro b-type natriuretic peptide; Hsc: hypersensitive cardiac; Pa02, partial pressure of oxygen; PaCO2, partial pressure of carbon dioxide; CT, computerized tomography; Ref., reference category for the calculation of hazard ratios in categorical variables with more than 2 categories.

**ACKNOWLEDGMENT**

The authors thank the CoCo-Neurosciences study group for their participation in the data collection

**Steering Committee** (Pitié-Salpêtrière Hospital, Paris): Cecile Delorme, Jean-Christophe Corvol, Jean-Yves Delattre, Catherine Lubetzki, Stephanie Carvalho, Aurelie Fekete, Sandrine Sagnes. **Scientific Committee** (Pitié-Salpêtrière Hospital, Paris): Bruno Dubois, Vincent Navarro, Celine Louapre, Tanya Stojkovic, Ahmed Idbaih, Charlotte Rosso, David Grabli, Ana Zenovia Gales, Bruno Millet, Benjamin Rohaut, Eleonore Bayen, Sophie Dupont, Gaelle Bruneteau, Stephane Lehericy, Danielle Seilhean, Alexandra Durr, Aurelie Kas, Foudil Lamari, Marion Houot, Vanessa Batista Brochard. **Principal investigators**: Pitié-Salpêtrière Hospital (Paris): Sophie Dupont, Catherine Lubetzki, Danielle Seilhean, Pascale Pradat-Diehl, Charlotte Rosso, Khe Hoang-Xuan, Bertrand Fontaine, Lionel Naccache, Philippe Fossati, Isabelle Arnulf, Alexandra Durr, Alexandre Carpentier, Stephane Lehericy, Yves Edel; Foch Hospital (Suresnes): Anna Luisa Di Stefano; Rothschild Hospital (Paris): Gilberte Robain, Philippe Thoumie; Avicenne Hospital (Bobigny): Bertrand Degos; Sainte-Anne Hospital (Paris): Tarek Sharshar; Saint-Antoine Hospital (Paris): Sonia Alamowitch, Emmanuelle Apartis-Bourdieu, Charles-Siegried Peretti; Saint-Louis Hospital (Paris): Renata Ursu; Tenon Hospital (Paris): Nathalie Dzierzynski; Charles Foix Hospital (Ivry): Kiyoka Kinugawa Bourron, Joel Belmin, Bruno Oquendo, Eric Pautas, Marc Verny. **Co-investigators**: Pitié-Salpêtrière Hospital (Paris): Cecile Delorme, Jean-Christophe Corvol, Jean-Yves Delattre, Yves Samson, Sara Leder, Anne Leger, Sandrine Deltour, Flore Baronnet, Ana Zenovia Gales, Stephanie Bombois, Mehdi Touat, Ahmed Idbaih, Marc Sanson, Caroline Dehais, Caroline Houillier, Florence Laigle-Donadey, Dimitri Psimaras, Agusti Alenton, Nadia Younan, Nicolas Villain, David Grabli, Maria del Mar Amador, Gaelle Bruneteau, Celine Louapre, Louise-Laure Mariani, Nicolas Mezouar, Graziella Mangone, Aurelie Meneret, Andreas Hartmann, Clement Tarrano, David Bendetowicz, Pierre-François Pradat, Michel Baulac, Sara Sambin, François Salachas, Nadine Le Forestier, Phintip Pichit, Florence Chochon, Adele Hesters, Bastien Herlin, An Hung Nguyen, Valerie Procher, Alexandre Demoule, Elise Morawiec, Julien Mayaux, Morgan Faure, Claire Ewenczyk, Giulia Coarelli, Anna Heinzmann, Perrine Charles, Tanya Stojkovic, Marion Masingue, Guillaume Bassez, Giorgia Querin, Vincent Navarro, Isabelle An, Yulia Worbe, Virginie Lambrecq, Rabab Debs, Esteban Munoz Musat, Timothee Lenglet, Virginie Lambrecq, Aurelie Hanin, Lydia Chougar, Nathalia Shor, Nadya Pyatigorskaya, Damien Galanaud, Delphine Leclercq, Sophie Demeret, Benjamin Rohaut, Albert Cao, Clemence Marois, Nicolas Weiss, Salimata Gassama, Loic Le Guennec, Vincent Degos, Alice Jacquens, Thomas Similowski, Capucine Morelot-Panzini, Jean-Yves Rotge, Bertrand Saudreau, Bruno Millet, Victor Pitron, Nassim Sarni, Nathalie Girault, Redwan Maatoug, Ana Zenovia Gales, Smaranda Leu, Eleonore Bayen, Lionel Thivard, Karima Mokhtari, Isabelle Plu; Sainte-Anne Hospital (Paris): Bruno Gonçalves; Saint-Antoine Hospital (Paris): Laure Bottin, Marion Yger; Rothschild Hospital (Paris): Gaelle Ouvrard, Rebecca Haddad, Paulina Cunha, Edouard Januel. Charles Foix Hospital (Ivry): Flora Ketz, Carmelo Lafuente, Christel Oasi, Vi-Huong Nguyen-Michel. **Other Contributors**: **Associated centers** (Lariboisière Hospital, Paris): Bruno Megabarne, Dominique Herve; **Clinical Research Associates** (ICM, Pitié-Salpêtrière Hospital, Paris): Amandine Hippolyte, Aurelie Fekete, Hugo Royer, Camille Minelli, Haysam Salman, Armelle Rametti-Lacroux, Alize Chalançon, Anais Herve, Florence Beauzor, Valentine Maheo, Christelle Laganot, Abel Grine, Marie Biet, Rania Hilab, Aurore Besnard, Meriem Bouguerra, Gwen Goudard, Saida Houairi, Saba Al-Youssef, Christine Pires, Anissa Oukhedouma, Katarzyna Siuda-Krzywicka, Tal Seidel Malkinson; (Saint-Louis Hospital, Paris): Hanane Agguini; (Foch Hospital, Suresnes): Hassen Douzane; **Data Manager** (ICM, Paris): Avigaelle Abitbol, Safia Said; **Statistician** (ICM, Paris): Marion Houot.
